# The Lucky Engine: Probabilistic Emergence and Persistence of Near-Maximum Dissipation States

**DOI:** 10.3390/e27070687

**Published:** 2025-06-27

**Authors:** Ralph D. Lorenz

**Affiliations:** Space Exploration Sector, Johns Hopkins University Applied Physics Laboratory, Laurel, MD 20723, USA; ralph.lorenz@jhuapl.edu; Tel.: +1-443-778-2903

**Keywords:** maximum entropy production, climate, optimality, combinatorics, frictional dissipation

## Abstract

A paradigm, wherein a nonequilibrium system has multiple modes of transport that can act in combination, permits the resolution of several difficulties with the notion of maximum entropy production (MaxEP or MEP). First, physical constraints, such as the density of the atmosphere or the planetary rotation rate, merely define the portfolio of modes that can be engaged by the system: physically impossible states cannot be selected. Second, with minimal sensitivity to how the system evolves, it is seen that there are simply more numerous quasi-steady microstates (combinations of modes) that are near the maximum of work output (or dissipation rate or EP) than there are far from it, and so it is more probable that the system will be observed to be near that maximum. Third, this paradigm naturally permits exploration of the system behavior when subjected to non-steady forcing. Finally, it provides a framework to explain when a system has ‘enough’ degrees of freedom to attain a maximum dissipation state, as opposed to the minimum dissipation state expected for certain constrained systems.

## 1. Introduction

Many physical systems are maintained in nonequilibrium configurations by the continuous supply of heat. The climate of the Earth, and planetary bodies in general, is a particularly significant example, with typically warmer conditions at low latitude where solar heating is stronger and cooler conditions at the poles. This temperature difference is mediated by a bewilderingly complex combination of heat transport processes in both the atmosphere and the oceans. Edward Lorenz (Edward Lorenz and the present author are to my knowledge unrelated, and sadly never actually met. The 1960 paper cited here [1] is not widely accessible, being a book chapter in the proceedings of a meeting in 1955. Note that it has quite different content from the almost identically titled but quite different 1955 journal paper by Lorenz [2], and should not be confused with it.), in 1955 [1], made the observation that the generation of mechanical energy by these heat transports on Earth was near the maximum it could possibly be.

Paltridge, in 1975 [3], examined an energy balance climate model (EBM) and found (more or less by trial and error [4]) that applying a criterion that an extremization of entropy exchange (later more intuitively expressed as a maximization of entropy production, or MaxEP) yielded good agreement with the Earth’s observed climate state [5,6].

The present author, in 2001 [7], made the observation that the climates of Mars and Titan, with very different circumstances (radiative forcing, atmospheric density, planetary diameter and rotation rate, etc.), also seemed to have equator-to-pole heat temperature gradients consistent with maximization of entropy production. The associated heat fluxes and effective diffusive transport coefficients varied by some two orders of magnitude.

This latter work triggered a repeat of many of the objections that were made in Paltridge’s work, most notably that the MaxEP principle appears to discard factors of known importance in planetary climate such as the density of the atmosphere and the rate of planetary rotation. Another confusing difficulty is that there was a well-established principle by Prigogine [8] that some systems (constrained, linear ones) *minimize* entropy production, and thus it is essential to lay out under what circumstances (if any) a *maximization* should emerge. While various arguments have been laid out on these questions—and the interested reader is referred to several reviews [9,10,11,12,13,14]—it can be said that the MaxEP notion has yet to find a secure footing.

The present paper aims to address this challenge by laying out a framework under which these aspects can be considered in a straightforward manner. In that respect, it aims to emulate the ‘two-box’ model [7]: while that work’s validity in supporting MaxEP can be questioned [15], it has the virtue that it is simple to understand [16,17].

## 2. Materials and Methods

**Figure 1:**
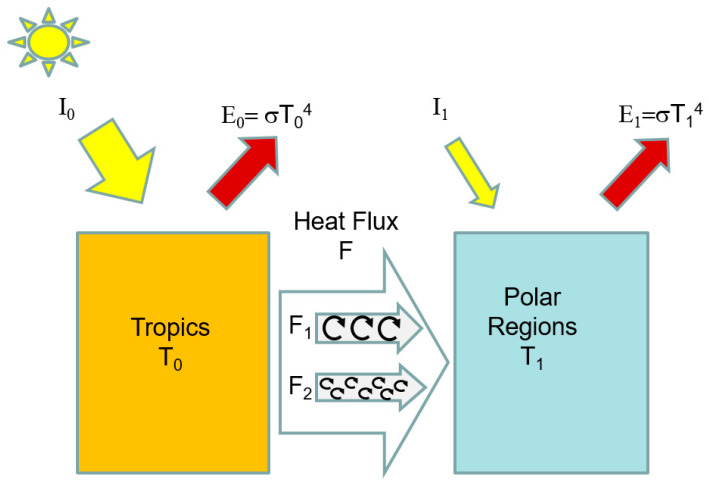
Two-box model of climate. Low- and high-latitude regions reach temperatures of T_0_ and T_1_, respectively, driven by solar fluxes I_0_ and I_1_. Heat is rejected to space via black body radiation fluxes E_0_, E_1_. Ocean and atmospheric heat transports F mediate the temperature difference and are driven by it (directly or indirectly). Here, two parallel component fluxes, F_1_ and F_2_, are shown, with the latter having a more turbulent, dissipative configuration (higher α).

We begin with the two-box energy balance climate model (EBM), notionally of the Earth’s climate. A low- and high-latitude zone each receive different amounts of sunlight, and each reject heat as a function of their temperature, T_0_ and T_1_, respectively. For the present-day Earth, the insolation I_0_ in the low-latitude box (taking into account the albedo) is about 300 Wm^−2^, while at higher latitudes, we have I_1_ = 170 Wm^−2^, following [7]. We ignore the greenhouse effect and assume that heat leaves the planet as thermal radiation from the two zones, with the outgoing emission related to temperature, e.g., E_x_ = σT_x_^4^ (many EBMs use a more or less empirical linear relationship instead, the results are not significantly different here). If heat is transferred between the boxes at a rate F (see Figure 1), then the system of equations is easily solved and the temperatures T_0_ and T_1_ are determined.

Clearly, a physically reasonable system (i.e., one where T_0_ > T_1_, so that heat is transported only down a temperature gradient) must have 0 < F < (I_0_ − I_1_)/2. In some EBMs, F is not unreasonably assumed to be proportional to the temperature difference, in effect acting as a diffusion process, i.e., F = k∆T with ∆T = (T_0_ − T_1_); but for simplicity of exposition, we will discuss only F in physical units. However, there is no universal ‘first principles’ means of determining F (or, equivalently, k), and they have generally been estimated in EBMs from empirical data.

**Figure 2:**
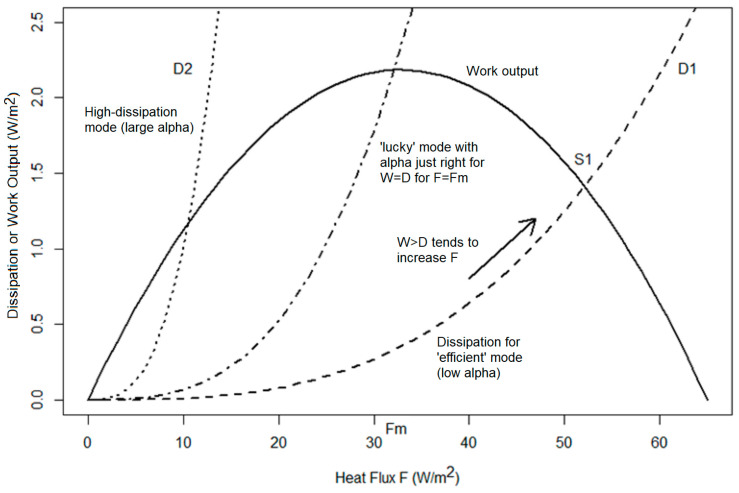
Work output rate W (solid line) as a function of the total heat flux F in the two-box model as described in the text (using I_0_ = 300 Wm^−2^, I_1_ = 170 Wm^−2^) and Figure 1. Increasing F from zero increases W at first, but eventually the heat-to-work efficiency drops as F brings the hot and cold temperatures to nearly isothermal conditions, so there is a maximum in W which occurs for F = F_m_, W = 0, for F = 0, and for F = 65 Wm^−2^. The frictional dissipation D is shown for an ‘efficient’ mode (e.g., D_1_, dashed line, with α_1_ = 10^−5^), and where W exceeds D, F would tend to increase (arrow) until an equilibrium (S_1_) is reached: provided α_1_ is low enough, and thus the D_1_ curve is shallow, S_1_ will be above the optimum. A less efficient (i.e., more dissipative) mode (e.g., D_2_, dotted line, α_2_ = 0.001) would cross the W curve below the optimum. A fortuitous mode with α = 6.6 × 10^−5^, or more likely some combination of F_1_ and F_2_, yields a net dissipation curve (dash-dot) which crosses the W curve right at the maximum.

Properties of interest are the Carnot limit on the rate of generation of mechanical work by this heat flux, i.e., W = F∆T/T_0_, and its entropy generation dS/dt = F(1/T_1_ − 1/T_0_). As noted by E. Lorenz [2,9], W tends to zero for small F and for large F, but has a maximum at some intermediate value, at which the Earth happens to sit (Figure 2). Simply put, at low F, there is no heat flux to convert into work, whereas for the maximum F, the system has been made isothermal (∆T = 0) and thus the efficiency with which heat transport is converted into work drops to zero: it is clear that there must be a maximum W at some intermediate value of F. The distinction of the F values for which W is a maximum and for which dS/dt is a maximum is not significantly different; also, since in a steady state, the frictional dissipation in the system must balance the generation of mechanical energy W, this steady configuration is often also termed one of ‘maximum dissipation’. Consideration of the fundamentals of thermodynamics suggests that the entropy production metric may well be the most fundamentally important (and in a general climate problem, sources of entropy production other than mechanical dissipation, such as that by mixing, are likely to be significant). However, here we again strive for clarity of exposition, so we consider in this paper only mechanical work and friction as concepts that are easier to understand. Note that in this paper, ‘work’ refers to the rate of generation of work (i.e., in W or W/m^2^). Similarly, in real planets, there is substantial vertical transport of heat and associated generation of work and entropy (e.g., [18,19]), whereas here we consider only the horizontal temperature contrasts and associated flows and generation. The arguments in the present paper are general and could be applied in the vertical dimension, but for clarity, we consider only the horizontal dimension (It has been remarked that it is probably not coincidental that the diffusion parameter k for the Earth has not only the same dimensions, but also the same numerical value (~1 W/m^2^/K) as the characteristic planetary average (radiative) entropy production, which is 0.5[(I_0_ − F) (1/T_0_) − (I_0_/T_S_) + (I_1_ + F) (1/T_1_) − (I_1_/T_S_)], where T_S_~6000 K is the temperature of the sun. Since T_S_ >> T_0_~T_1_, this reduces to approximately 0.5(I_0_ + I_1_)/0.5(T_0_ + T_1_), or the average solar flux divided by the average temperature. This same ‘coincidence’ is true for Titan, even though the respective values are almost two orders of magnitude smaller (~0.01 W/m^2^/K), contrary to what one would expect from scaling Earth’s k value to Titan’s pressure and rotation rate. In the EBM literature, k is often given the symbol D, although in this paper, we preferred to avoid confusion with dissipation).

Returning to the 1-D latitudinal EBM, the crux of the problem is that F is unknown. Even for a pure fluid on a homogenous and flat rotating planet, F cannot be uniquely deduced from first principles, and of course the real Earth has heat transports through complex flows in both the atmosphere and the ocean, with heat conveyed in part by the latent heat of water as well as by sensible heat in the two main fluids.

If only a single transport mode is present (with some dissipation function D(F), which is likely nonlinear (e.g., D = αF^3^, see later, although nonlinearity is not essential to the argument), then for a given set of boundary conditions (I_0_, I_1_) which determines the work output W(F), it follows that a steady state (e.g., S1 in Figure 2) occurs where the curves cross (W = D). The system is then uniquely specified (and such a constrained system recalls the minimum entropy production arguments of Prigogine, Jaynes, and others e.g., [20,21]). It is only if the dynamics of the transport mode (α) are ‘lucky’ such that W = D happens to be at the maximum work output that the maximum dissipation state is found.

**Figure 3:**
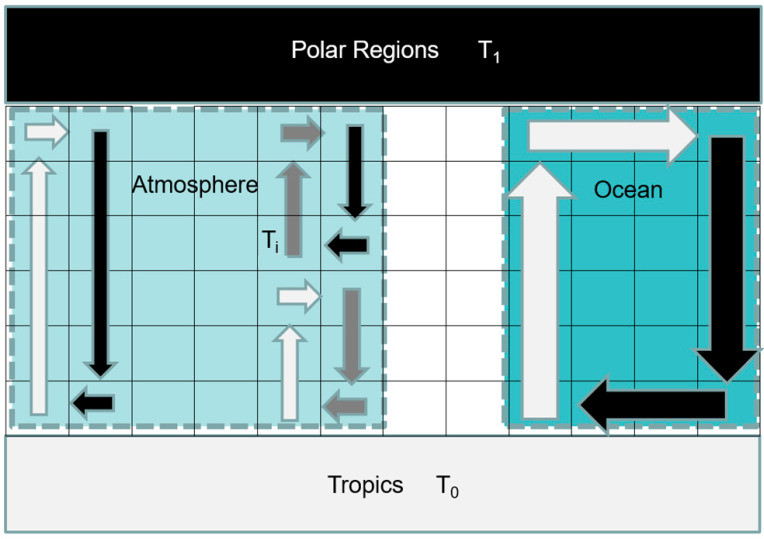
Flow configurations. The leftmost and middle flow patterns illustrate two configurations of flow that may operate in parallel in the atmosphere: arrows denote fluid motion, with the color indicating temperature (lighter grey corresponding to higher temperatures). The leftmost flow pattern, notionally F_0_, is a large-scale circulation that is efficient in having little dissipation and conveys parcels of air carrying heat associated with the full equator-to-pole temperature contrast, while a more turbulent eddy mode (middle, F_1_) has more frictional shear and may involve cells moving parcels of air at intermediate temperatures (T_i_) that are less thermally efficient at heat transport than if parcels were moved from equator to pole. Ocean heat flows operate on Earth as well, with a very different column mass and heat capacity, and much lower velocities, than in the atmosphere. The geometry and symbology of this graphic are not intended to accurately portray any features of Earth’s actual fluid motions, only to illustrate that multiple transport paths may exist in parallel with different dissipation characteristics (α).

Only with very mechanically restricted arrangements is it specified, however, since a fluid can take many paths, each with different α (or indeed, there may be multiple fluids present—see Figure 3). The argument I advance here essentially notes that this underdetermination can be resolved statistically—it is the very fact that the heat transport is the result of the combination of many elements that allows its net effect to be predictable, just as conventional thermodynamics allows the prediction of large-scale properties of a gas without requiring knowledge of the energies of every individual molecule (an analogy noted by Paltridge [1] and Dewar [22,23]). Some elements of the paradigm proposed here (notably that the matching of maximum work output to maximum dissipation can be achieved by the combination of low- and high-dissipation heat transports) were articulated briefly in, e.g., a commentary article in 2003 [24], but the present paper permits a more comprehensive development. In fact, elements of the paradigm were actually articulated by E. Lorenz [1] in his observation that the Earth’s atmosphere seems to be in a state of maximal generation of available potential energy (APE). This energy (or work) is generated by the radiative deposition of heat at the ‘hot’ end of the engine. In the steady state, this is equivalent to maximum mechanical dissipation, since the APE becomes kinetic energy which is then dissipated by friction and viscosity. Aspects of ‘how’ the system accesses and reaches the maximum were noted in that work, and by [5,6], and in a review by Ozawa et al. [9], but the *combinatorial* emergence of the optimum was not described.

## 3. Results

Let us assume that the heat transport F is the sum of two components, F_1_ and F_2_, corresponding to some large-scale overturn of the atmosphere and some smaller-scale eddies (or, perhaps, F1 might correspond to ocean transport and F2 the atmosphere—see Figure 3). If we consider sensible heat transport, then these two terms will be associated with corresponding flow velocities V_1_ and V_2_, and there will be associated frictional dissipation D_x_~V_x_^3^ (since the drag force per unit area is proportional to V^2^), or since F is proportional to V, we can write D_x_ = α_x_F_x_^3^ with α_x_ as a constant for that transport mode x = [1, 2, …]: α_x_ encodes the density of the fluid, the surface roughness which controls the effective drag coefficient, and some geometric factor describing the flow pattern, e.g., the size of the characteristic eddy. Clearly, since a set of small eddies has a more tortuous path from low to high latitude than a large-scale overturn, then α_1_ >> α_0_. Schematically, such flows are shown in Figure 2; some aspects of these flows are discussed in the MaxEP context by Kleidon and colleagues [25].

So far, we have not simplified the problem—we just introduced another unknown. In steady state, we can choose F_1_ and F_2_ to be any arbitrary values up to

F_1_ + F_2_ ≤ (I_0_ − I_1_)/2. (1)

The work output, W, varies as a function of F = (F_1_ + F_2_), with some maximum value and an intermediate F as discussed earlier (solid line in Figure 2). Now, if we set F_2_ = 0 and imagine that the large-scale transport F_1_ has a rather weak dissipation D_1_ = α_1_F_1_^3^, which is a monotonically increasing function of F_1_ (dashed line in Figure 2), the D(F) and W(F) curves cross at a position determined by the various parameters, notably α_1_, for an F value F_s1_ large than that for the peak F_m_. At this crossing point, mechanical energy generation and frictional dissipation are equal, so the system has a possible steady state. The constraints on the system (embodied in α_1_) are such that the system can in principle access the configuration F_m_; however, the dissipation here is lower than the mechanical energy generation rate, so the system would tend to speed up, moving away from the peak. These aspects of system behavior were discussed previously by E. Lorenz, Ozawa, and others.

Contrariwise, we may imagine that F_1_ can be set to zero, and F_2_ can vary. However, the F_2_ transport mode is much less efficient in the sense that there is a much higher frictional dissipation per unit heat transport (i.e., α_2_ >> α_1_) and the D_2_(F) function grows much steeper than in the previous example. Thus, the intercept of this curve (dotted line in Figure 3) with the W(F) mechanical energy generation curve (solid line) occurs at a much lower value of F, i.e., to the left of the peak in W. In this case (i.e., if the F_2_ mode was the only one accessible to the system due to dynamical constraints on density, rotation and so on, as encoded in α_2_), the system cannot access the maximum dissipation state, because W(F_m_) < D(F_m_). As soon as the system spins up beyond the crossing point, W < D and mechanical energy is lost, tending to reduce F_2_ so F_m_ cannot be reached.

It is easy to imagine that one could have hybrid configurations with F_1_ > 0 and F_2_ > 0, which will have a net dissipation intermediate between these two end members. Algebraically, we could choose combinations of F_1_ and F_2_ that have dissipations equal to the work output for F above, below, or exactly at the optimum F_m_. We could generalize further to some arbitrarily large number of different modes.

We can see, then, that as long as one or more modes exist with dissipations both suitably low and high, then it is theoretically possible to have a MaxEP steady state. Since arbitrarily tortuous flow paths can be imagined, it is always possible to obtain suitably high dissipation modes, thus, the necessary condition becomes that *at least one permitted configuration has dissipation weaker than that needed to reach the optimum*. However, this does not explain why a given system should find this optimum.

**Figure 4:**
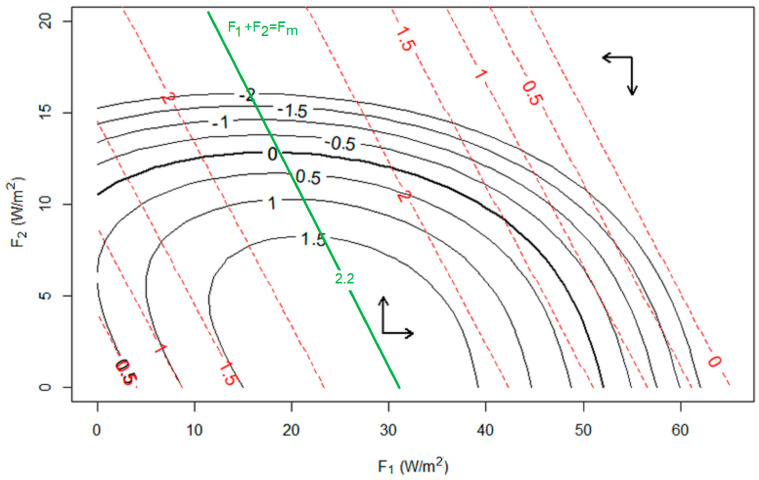
The work dissipation balance of Figure 2 is shown in the state space of (F_1_, F_2_) for a two-mode system. The dashed red contours are the work output, which has an optimum of ~2.2 Wm^−2^ (green line) for F_1_ + F_2_~F_m_~32 Wm^−2^. The solid black contours show the imbalance of kinetic energy generation minus dissipation, (W − D): the thick zero contour of this quantity corresponds to a steady state where these quantities are balanced, and thus the system will likely evolve only slowly near this line. Away from the line, the exact evolution of the system will depend on the dynamics imposed on the transfer of energy into and between the modes, but must be in the quadrants identified by the arrows (i.e., when W − D > 0 as in the lower center of the graph, kinetic energy can increase, so F_1_ or F_2_ must increase, or both). The dissipation of the F_2_ mode is so high that above F_2_~10 Wm^−2^, the dissipation increases rapidly. The red W = 0 contour at right corresponds to the practical limit F_1_ + F_2_ = 65 Wm^−2^ set by the radiative constraint (I_0_ − I_1_) = 130 Wm^−2^, and only states to the lower left of this line are physical. Of note is that much of the (W − D) = 0 steady state line is within the W > 2 Wm^−2^ contour, near the optimum.

To address this question, consider the set of available states (for discussion purposes, to make this a finite set, let us assume that the heat transports in the two modes must have integer values in Wm^−2^). This then defines a triangular set of points in the state space of (F_1_, F_2_)—see Figure 4. Some of these states have dissipation higher than the work output of the system in that configuration (i.e., W < D), in which case the system would be expected to spin down quickly, i.e., change to a lower energy state with lower F_1_, lower F_2_, or both. The opposite evolution is expected when W > D.

However, some configurations may have dissipations rather close to the work output—in such configurations, one may imagine that the system will change state only slowly since kinetic energy is being added at nearly the same rate W as that at which it is being removed by D. However, the time evolution of F_1_, F_2_, etc., depends on the actual dynamics of the system, specifically how kinetic energy is transferred to and from the different modes F_1_ and F_2_.

In general, one could imagine a set of couplings of the form.

dF_1_/dt = β_1_(W − D_1_) − γ_1_(F_1_ − F_2_) (2)

dF_2_/dt = β_2_(W − D_2_) + γ_2_(F_1_ − F_2_) (3)

The parameters β_1_ and β_2_ describe how available potential energy (i.e., the temperature difference) drives the motions, while parameters γ_1_ and γ_2_ describe how friction transfers momentum from F_1_ to F_2_. The dynamical interpretation is that coupling exists between the different modes; for example, energy will flow from a large-scale circulation to smaller eddies in the familiar Kolmogorov cascade, or wind stress at the sea surface will cause ocean currents to develop. In the first instance, the dissipation per unit heat transport increases (since the smaller-scale eddies have more shear than the large-scale flow), whereas in the second, the dissipation per unit heat transport decreases, because the density and heat capacity of the ocean water column is much larger than that of the ocean (for the present-day Earth, at least). Physical models, typically with empirically derived parameters (such as the sea surface drag coefficient) can be developed for these couplings, and this is how general circulation models typically are constructed (we may incidentally recall that the system of Equations (2) and (3) has some similarities with the convecting system, whose nondeterministic behavior was noticed by Lorenz [26], a discovery—‘chaos’—that become altogether more famous than his work on the extremal state of the climate).

The details needed to accurately model such modes and couplings are not always available, however. Ocean circulation on Earth is driven, for example, to some extent by the equator-to-pole temperature difference (although warm surface waters must be mechanically mixed downwards by difficult-to-model effects of, e.g., hurricanes, in order to provide the buoyant force driving the large-scale circulation). Large-scale wind stress over the ocean is also an important factor. We could reasonably posit that the acceleration of the ocean circulation mode has two terms, one related to the temperature difference (i.e., β), and one to the speed of the atmospheric mode, i.e., γ (Strictly, a frictional coupling would imply that the drag force per unit area accelerating the ocean current would be proportional to the square of the difference between surface wind speed and the ocean surface current. However, since the ocean column has such a large heat capacity, the ocean speeds are small relative to the wind and can be ignored. In this respect, the kinetic energy transfer is effectively unidirectional. Planetary environments, e.g., with very shallow oceans and with very dense atmospheres, could be contrived where two-way coupling would need to be considered.). Note that if these couplings are too strong, the system loses degrees of freedom (e.g., forcing F_1_ and F_2_ to be strongly connected is algebraically equivalent to choosing an intermediate k value and thus explicitly determining the steady state W = D crossing point).

The essence of the MaxEP hypothesis is that these details *do not need to be known*. What matters is that at least one mode exists with a dissipation less than the maximum and at least one exists with a dissipation more than it, and some coupling in both directions allows the system to evolve. In the planetary climate context, satisfaction of the first condition is not always met (e.g., the atmosphere is too thin, or the planet rotates too fast, etc., and it is the lack of a statement about this condition that caused dynamical meteorologists to be deeply uneasy with the MaxEP notion). In situations where the first condition is met, it seems that the second will always be met too, simply as a result of the character of fluid turbulence (e.g., [27]). One can always invoke ever smaller eddies to soak up excessive kinetic energy by viscosity, per Richardson’s poem.

**Figure 5:**
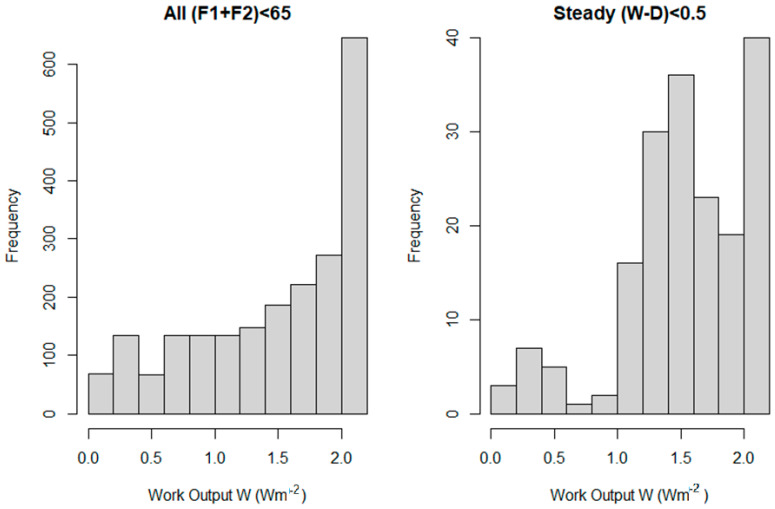
The relative likelihood of W values across the physical state space shown in Figure 4 (i.e., those points with F_1_ = (0, 1, 2, …, 65) and F_2_ = (0, 1, 2, …, 65)), and F_1_ + F_2_ < 65 Wm^−2^ is shown on the left. However, when we select points that are close to steady state (arbitrarily, abs(W − D) < 0.5 Wm^−2^), we find points near the optimum are much more strongly represented.

It follows, then, that if the system can evolve throughout the state space, then the likelihood of some observed property (such as near-maximum dissipation) will depend on the fraction of the state space showing that property. Figure 5 shows the relative frequencies of dissipations in the state space of Figure 4 (i.e., assuming the system resides for equal amounts of time on each point in the allowed space)—higher frequencies are seen for higher dissipations. However, the propensity to observe near-maximum dissipations is much higher when we consider only those points which are near steady state, i.e., with W~D. There are simply proportionately more such states when the dissipation is higher, because there are more possible combinations of (D_1_, D_2_) that sum to a higher number (W) than there are that sum to a smaller one.

More generally then, the dimensionality of the state space is higher than two, and the location in that space is described by a large vector (F_1_, F_2_, F_3_…F_n_) of (assumed positive) quantities describing the vigor of each of the modes. The net heat transport is evaluated by a weighted sum of these values—if the vector was specified as mass flows, then the weights would incorporate factors that encapsulate the transport properties of each mode (e.g., the horizontal size of eddies, the column mass and heat capacity of the atmosphere, and so on), although more generally, additional factors, such as latent heat, could be included. The dissipation is a weighted sum of some probably nonlinear (notionally cubic, in the classic drag formulation noted earlier) functions of the heat transports. The propensity of the system to be close to a maximum in dissipation simply arises from the number or density of microstates for which dissipation is α_1_F_1_^3^ + α_2_F_2_^3^ + α_3_F_3_^3^…+ α_n_F_n_^3^~W, which is higher for large W.

**Figure 6:**
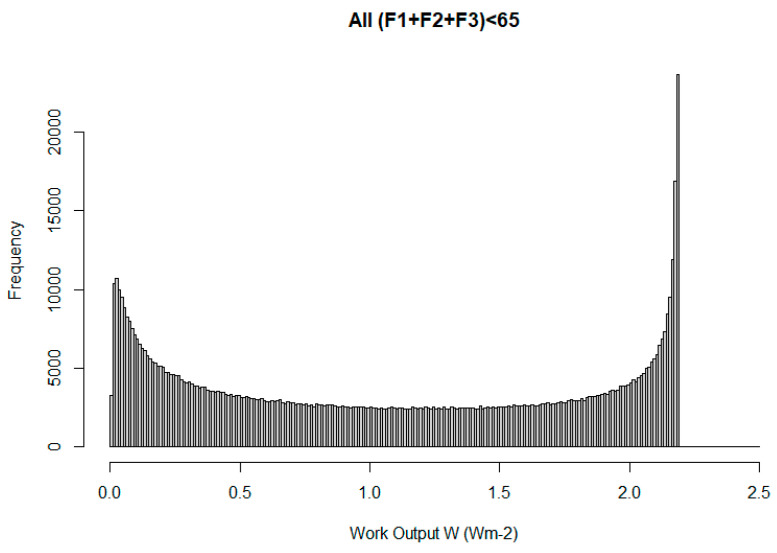
Simply as a result of the larger number of permitted combinations, the observed work output peaks at the maximum value. For this particular example, a small secondary peak near zero appears, but this is an artifact of the chosen F distributions.

As another example, then, we can consider a more general three-mode system, with α values of 0.01, 0.001, and 0.0001, and allowable F values for each having distributions uniform in the logarithm of F in the range 0.01–65 Wm^−2^. Although this choice is entirely arbitrary, it avoids the restriction of integer values in the previous example (which in turn biased the order of magnitude that resulted). Again, however, it is seen (Figure 6) that the distribution of W values peaks at the maximum, and indeed, this results without requiring the selection of the steady state (W~D). The principle is rather general.

Now, while the explicit dynamics of the system could be specified by couplings in a higher-dimensional analogy of Equations (2) and (3), one could instead imagine evolution via a conditional random walk, similar to the search algorithm of an optimizer. A rational physical rule would be, for example, to simply increment one coordinate F_i_ (i randomly chosen from 1 to n) if the dissipation is less than the work output such that the overall kinetic energy increases, or decrement it if dissipation exceeds work (i.e., spin down). It is easy to see that such an evolution would naturally spin up from rest and will tend to fluctuate about a steady state, which will most frequently be near the maximum of total dissipation.

## 4. Discussion

The framework outlined here is natural and general and lays out how additional degrees of freedom contribute to permitting the maximum dissipation state to emerge and persist.

The notion that the existence of a lowest dissipation mode of heat transport that allows the maximum dissipation state to be attained is only permitted (with some reasonable assumptions about planetary flow configurations) for certain minimum atmospheric column densities and some maximum planetary rotation rates was examined in [17]. The idea in the previous section that other less efficient modes can then spin up to ‘absorb’ the excess generated work is hinted at in the GCM work [28], which showed that as the resolution of a GCM was increased, the entropy production by flows increased asymptotically.

Some state-space behaviors of a numerical ocean circulation model were noted in [29]. It was found that perturbing the model tended to cause evolution to steady states of higher entropy production (i.e., local attractors in state space), except where the perturbation was so large as to fundamentally destroy the pre-existing flow configuration, i.e., to make a jump so far in state space that the local optimum was ‘lost’. This behavior seems consistent with the paradigm laid out in the present paper.

Finally, we may note that Paltridge [30] attempted to argue that fluctuations in the system would progressively ratchet the system to a state of higher dissipation. An electrical circuit with a diode to embody this tendency was offered as an illustration, but the work conceded that it was not “a perfect analog” and admitted that the author had not been able to prove the behavior in a general way.

Thus, a note on vocabulary is in order. It was often said in the MaxEP literature that the system ‘chooses’ a state of MaxEP, or that MaxEP may act as a ‘selection principle’. These words run afoul of the aversion to teleology that was also encountered, for example, in Lovelock’s Gaia principle [31]. It has been shown here that the system makes no “choice”, it is free to meander among different states. However, for any reasonable physical dynamic, the system is simply more likely to be in or near the maximum at any moment than to be far from it. In that sense, MaxEP is a close analogue of conventional statistical thermodynamics, which allows for (indeed, predicts) transient deviations from a state of maximum entropy and leads to Brownian motion or other fluctuations. The work of Dewar [32] elucidating this analogy is particularly notable.

## 5. Conclusions

**Figure 7:**
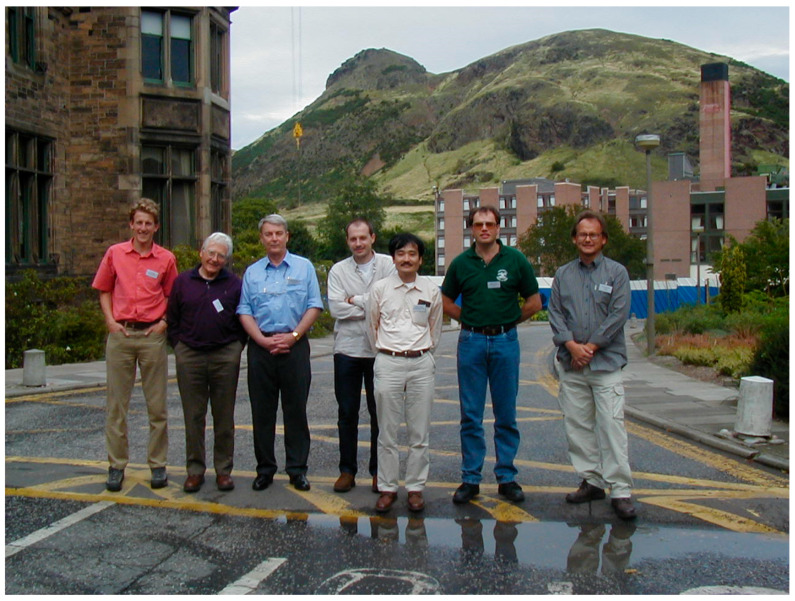
A workshop “Beyond Daisyworld” was convened in Edinburgh, Scotland in September 2002 at which the author met and had fruitful discussions with many of the pioneers of the Maximum Entropy Production hypothesis. From left to the right are Tim Lenton (who convened the meeting), James Lovelock, Garth Paltridge, Toni Pujol, Hisashi Ozawa, the author, and Roderick Dewar.

This paper attempted to make clearer a concept whose essence can be discerned in a number of previous works by others (see Figure 7), namely that the observation of the Earth’s climate and others may be in a maximum dissipation state is not the result of some fortunate tuning of the dynamics of particular heat transport modes, but emerges statistically as the result of the fact that there are simply more numerous steady state combinations of modes near the maximum dissipation state than there are away from it. It is hoped that this combinatorial perspective may help the MaxEP paradigm find useful applications.

## Data Availability

No new data were created or analyzed in this study. Data sharing is not applicable to this article.
